# Establishing Machine Learning Models to Predict Curative Resection in Early Gastric Cancer with Undifferentiated Histology: Development and Usability Study

**DOI:** 10.2196/25053

**Published:** 2021-04-15

**Authors:** Chang Seok Bang, Ji Yong Ahn, Jie-Hyun Kim, Young-Il Kim, Il Ju Choi, Woon Geon Shin

**Affiliations:** 1 Department of Internal Medicine Hallym University College of Medicine Chuncheon Republic of Korea; 2 Institute for Liver and Digestive Diseases Hallym University Chuncheon Republic of Korea; 3 Institute of New Frontier Research Hallym University College of Medicine Chuncheon Republic of Korea; 4 Division of Big Data and Artificial Intelligence Chuncheon Sacred Heart Hospital Chuncheon Republic of Korea; 5 Division of Gastroenterology Department of Internal Medicine University of Ulsan College of Medicine, Asan Medical Center Seoul Republic of Korea; 6 Division of Gastroenterology Department of Internal Medicine Gangnam Severance Hospital, Yonsei University College of Medicine Seoul Republic of Korea; 7 Center for Gastric Cancer Research Institute and Hospital National Cancer Center Goyang Republic of Korea

**Keywords:** early gastric cancer, artificial intelligence, machine learning, endoscopic submucosal dissection, undifferentiated, gastric cancer, endoscopy, dissection

## Abstract

**Background:**

Undifferentiated type of early gastric cancer (U-EGC) is included among the expanded indications of endoscopic submucosal dissection (ESD); however, the rate of curative resection remains unsatisfactory. Endoscopists predict the probability of curative resection by considering the size and shape of the lesion and whether ulcers are present or not. The location of the lesion, indicating the likely technical difficulty, is also considered.

**Objective:**

The aim of this study was to establish machine learning (ML) models to better predict the possibility of curative resection in U-EGC prior to ESD.

**Methods:**

A nationwide cohort of 2703 U-EGCs treated by ESD or surgery were adopted for the training and internal validation cohorts. Separately, an independent data set of the Korean ESD registry (n=275) and an Asan medical center data set (n=127) treated by ESD were chosen for external validation. Eighteen ML classifiers were selected to establish prediction models of curative resection with the following variables: age; sex; location, size, and shape of the lesion; and whether ulcers were present or not.

**Results:**

Among the 18 models, the extreme gradient boosting classifier showed the best performance (internal validation accuracy 93.4%, 95% CI 90.4%-96.4%; precision 92.6%, 95% CI 89.5%-95.7%; recall 99.0%, 95% CI 97.8%-99.9%; and F1 score 95.7%, 95% CI 93.3%-98.1%). Attempts at external validation showed substantial accuracy (first external validation 81.5%, 95% CI 76.9%-86.1% and second external validation 89.8%, 95% CI 84.5%-95.1%). Lesion size was the most important feature in each explainable artificial intelligence analysis.

**Conclusions:**

We established an ML model capable of accurately predicting the curative resection of U-EGC before ESD by considering the morphological and ecological characteristics of the lesions.

## Introduction

Endoscopic submucosal dissection (ESD) is indicated for the treatment of patients with early gastric cancer (EGC) satisfying prespecified criteria, including histology, according to the differentiation, specific lesion size, morphology, and whether ulcers are present or not in the target lesion. The long-term prognosis following ESD for cases of EGC meeting the ESD criteria (achievement of curative resection) is comparable to that achieved with surgical resection [1,2]. In the context of histology, the undifferentiated type of EGC (U-EGC) generally refers to poorly differentiated adenocarcinoma, signet-ring cell carcinoma, or mucinous adenocarcinoma [3,4]. Although U-EGC is included among the expanded indications of ESD (mucosal U-EGC<2 cm without ulceration and without evidence of lymphovascular invasion), the rate of curative resection in U-EGC has remained very low—reported previously as 61.4% in a meta-analysis and 36.4% in a nationwide cohort study in Korea [5,6]. This implies that an unmet need persists regarding the accurate prediction of curative resection in U-EGC (ie, difficulty in adopting a precise ESD indication). Therefore, proper candidate selection prior to ESD is important.

Endoscopists predict the probability of curative resection by considering the size and shape of the lesion and whether ulcers are present or not. These components together compose the indications of ESD. In addition, lesion location, which can suggest the expected technical difficulty during the procedure and hint at the general condition of the patient, is also considered prior to conducting ESD. However, U-EGC has distinctive growth patterns relative to differentiated-type EGC [3,4,6,7]. U-EGC is known to extend laterally along the proliferative zone in the intermediate layer of the mucosa (subepithelial spreading), and the development pattern from the intermediate layer could lead to nonexposure to the surface mucosa, limiting the precise measurement of lesion size [5,8]. Subepithelial-spreading signet-ring cell carcinoma is more prevalent than the epithelial-spreading type in cases with background atrophy or intestinal metaplasia of the gastric mucosa [9,10]. Further, ESD of poorly differentiated adenocarcinoma presents a stronger association with submucosal invasion relative to that of signet-ring cell carcinoma [6]. Although adopting a precise indication is a key ability of endoscopists, U-EGC itself is a risk factor for a greater out-of-indication rate, leading to noncurative resection [11,12].

With the extensive production and collection of ongoing medical data, the application of artificial intelligence has been attempted in clinical practice [13]. Machine learning (ML) is a mathematical artificial intelligence algorithm automatically built from given data to predict precise outcomes in uncertain conditions without being explicitly programmed [14]. Examples of ML include Bayesian inferences, decision trees, support vector machines, deep neural networks, or ensemble methods (bagging or boosting) [14]. In short, ML is a type of applied statistical technique and is characterized by high accuracy. We aimed to establish an ML model to better predict the possibility of curative resection in U-EGC prior to ESD.

## Methods

### Ethical Statement

This study was approved by the Institutional Review Board of the Chuncheon Sacred Heart Hospital, Korea (no. 2020-07-019). It adhered to the principles expressed in the Declaration of Helsinki.

### Data Sets

A nationwide cohort of 2703 U-EGCs treated by ESD (n=967) or surgery (n=1736) from 2006 to 2015 composed the training and internal validation groups. Eligible subjects were retrospectively enrolled from 18 university hospitals in Korea. Separately, an independent data set involving the Korean ESD registry with 275 U-EGCs and an Asan medical center data set with 127 U-EGCs treated by ESD were used for external validation. Subjects in the Korean ESD registry data set were retrospectively identified from 8 institutions of Korea [6], having been treated with ESD from 2006 to 2015, while subjects in the Asan medical center data set were treated by ESD from 2007 to 2013. All these data sets were mutually exclusive.

### ML Models

**Figure 1:**
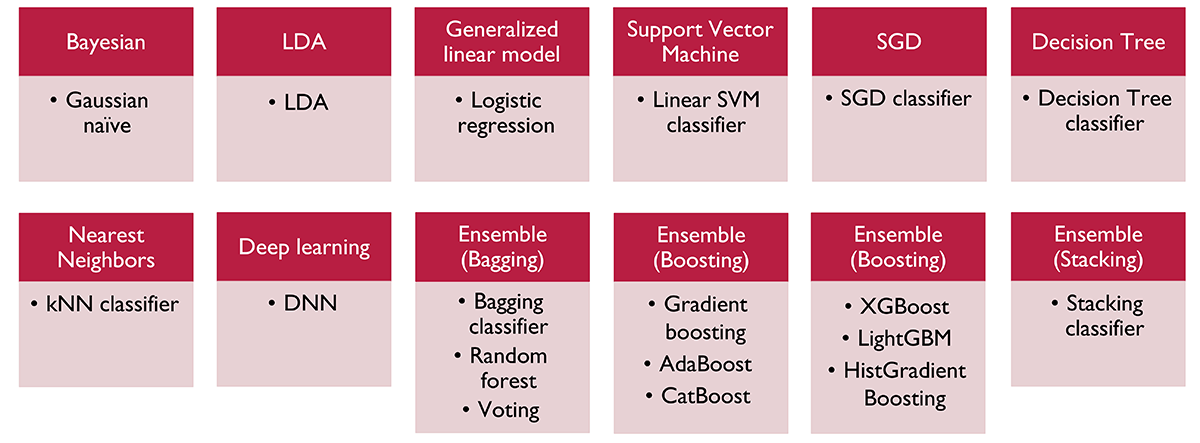
Machine learning classifiers used in this study. AdaBoost: adaptive boosting; CatBoost: categorical boosting; DNN: deep neural network; HistGradientBoosting: histogram-based gradient boosting; kNN: k-nearest neighbors; ML: machine learning; LightGBM: light gradient boosting machine; LDA: linear discriminants analysis; SGD: stochastic gradient descent; SVM: support vector machine; XGBoost: extreme gradient boosting.

All the currently available types of supervised ML classifiers were tested for the establishment of a curative resection prediction model in U-EGC. In total, 18 ML classifiers were assessed, including naïve Bayes in Bayesian inferences, linear-discriminant analysis, logistic regression in generalized linear modeling, linear support vector machine, stochastic gradient descent, decision tree, k-nearest neighbors, deep neural networks, bagging ensemble methods (bagging classifier, random forest, and voting classifier), boosting ensemble methods (gradient boosting, adaptive boosting, categorical Boosting, extreme gradient boosting [XGBoost], light gradient boosting machine, histogram-based gradient boosting), and a stacking ensemble method (stacking classifier). The Gaussian Naïve Bayes classifier is a model based on the Bayes’ theorem encompassing the assumption that there is independence between the features. A generalized linear model is the extension of a linear model set up to include cases where the dependent variable is not normally distributed. We adopted the logistic regression classifier for this study. The support vector machine is a model that defines a decision boundary (hyperplane), that is, a reference line for classification. The stochastic gradient descent is a model for linear classifiers under convex loss functions such as support vector machine and logistic regression [15]. The decision tree is an algorithm that automatically finds rules in the data and creates tree-based classification rules. k-nearest neighbors is a classification or clustering algorithm that relies on distance metrics measures for similarity. Deep neural networks refer to an artificial neural network with multiple hidden layers between the input and output layers that learns from input data and optimizes the output classification with mathematical calculations. Ensemble algorithms combine multiple classification models to achieve better performance and can be classified as either bagging, boosting, or stacking methods. Bagging is a parallel ensemble method that fits individual random samples of the data set and aggregates the predictions of each model for the final classification (bootstrap aggregation) [15]. This meta-estimator can reduce the variance of each classification model by introducing randomization for the model establishment and then creating an ensemble out of it. As such, bagging reduces overfitting of the ML model [15]. Separately, boosting algorithms attempt to conduct ensemble modeling sequentially by learning from the errors of the previous model and updating the weight of subsequent models to optimize the loss functions and reduce the overall bias. In contrast with learning from homogenous weak models in the bagging and boosting algorithms, stacking algorithms learn from heterogeneous models, creating a meta-model for the final classification. For the current ML analysis of this study, we used bagging classification, random forest, and voting classification for the bagging ensemble methods and gradient boosting, adaptive boosting, categorical boosting, XGBoost, light gradient boosting machine, and histogram-based gradient boosting for the boosting methods. For the stacking algorithm, we chose stacking classification. All the ML classifiers were imported from the scikit-learn package version 0.23.2 using the Python programming language (version 3.8.5, Python Software Foundation). Figure 1 shows the types of ML classifiers examined in this study.

### Variables, Primary Outcome, and Data Splitting

A total of 18 ML classifiers were used for the establishment of prediction models of curative resection with the following variables: age; sex; location, size, and shape of the lesion; and whether ulcers were present or not. The primary outcome was the accuracy of the established ML models for the prediction of curative resection with the given variables of the lesions. Thus, the main metric was the classifying accuracy. Each data set was prepared in the .csv file format. After uploading .csv files to the Google Colaboratory analysis platform, 2703 U-EGC data points were randomly split into training and internal validation sets according to a ratio of 9:1.

### Definitions of the Variables

Among the variables used in this study, patient age and the size of the lesion were the continuous variables and the others were considered as categorical variables. The location of the lesion was categorized by both longitudinal location (lower-third, mid-third, and upper-third) and circular location (lesser curvature, greater curvature, posterior wall, and anterior wall). The shape of the lesion was defined in accordance with the Japanese classification: elevated, flat, or depressed according to the morphological characteristics. According to this system, type I (protruded) and type IIa (superficial elevated) were considered as elevated, type IIb (flat) and type IIc (superficial depressed) were considered as flat, and type III (excavated) was considered as depressed [4]. Curative resection was defined as complete resection of U-EGC with a diameter of 2 cm or less and a lesion confined to the mucosa, with negative lateral and deep resection margins and lymphovascular invasion. Noncurative resection referred to cases in which the resected lesion did not fulfill these criteria.

### Statistical Analysis and Explainable Artificial Intelligence

Continuous variables were expressed as mean (SD) and categorical variables were expressed as numbers and percentages. Descriptive synthesis was conducted to reveal the baseline characteristics of the training and internal validation data set and external validation data set. To add to the interpretability of the established ML model, we performed an explainable artificial intelligence analysis. To elucidate the variables associated with lesions either accurately or inaccurately determined by the ML model, univariable analysis was conducted (Student *t* test and Fisher exact test for continuous and categorical variables, respectively). A two-tailed *P* value of less than .05 was adopted as the threshold for statistical significance. These analyses were performed using SPSS version 24.0. (IBM Corporation). Additionally, a feature importance (or permutation importance) analysis was completed to reveal which variables primarily contributed to the model’s decision process [16,17]. This assessment measures the predictive error when a certain feature value is randomly shuffled; therefore, insignificant features do not affect the performance of the model [15]. Feature importance is measured by the F-score, which represents the ratio between the explained and the unexplained variance [17]. A decision process tree was plotted to visualize the step-by-step process of the decision making of the established ML model using the Graphviz package (version 0.14.1; AT&T Labs Research). A partial-dependence plot tool box (version 0.2.0) in the scikit-learn package to visualize the important features for the ML model was adopted and the target plot and interaction plot were visualized [18,19]. A Shapley additive explanations (version 0.35.0) analysis is an approach used to explain the output of any ML model using Shapley values and the degree of independence between features. The Shapley value expresses how much each feature contributes to creating the overall performance and represents feature importance while maintaining consistent and locally accurate additive feature attribution for a particular prediction [20].

## Results

### Characteristics of the Training, Internal Validation, and External Validation Data Sets

The training and internal validation data sets contained not only endoscopically resected cases but also surgically removed cases of U-EGC. The first external validation data set was composed of a nationwide cohort of cases of ESD performed for U-EGC, while the second external validation data set consisted of cases of ESD performed for U-EGC from a single hospital with the largest degree of ESD experience to date in Korea. Therefore, the included data sets were marked by different clinical characteristics. Table 1 presents the detailed clinical characteristics of the included lesions in this study. A male sex predominance was consistently observed in all data sets. Patient age ranged from 64.1 (SD 13.0) years to 67.8 (SD 12.0) years. In the context of endoscopic findings, the lower-third part in the longitudinal location (2069/2703, 76.5% and 214/275, 77.8%) and lesser curvature in the circular location (97/275, 34.5% and 945/2703, 34.9%) were the most frequent lesion positions in the training and internal validation dataset and first external validation data set, respectively. Meanwhile, the mid-third part was the most frequent lesion location in the longitudinal location (61/127, 48.1%) for U-EGC in the second external validation data set. The mean endoscopic size of the included lesions ranged from 21.7 (SD 12.5) mm to 27.9 (SD 16.2) mm. Depressed lesions (type IIc) were observed as the most frequent morphological type in the training and internal validation data set and second external validation data set (1762/2703, 65.2% and 62/127, 48.8%, respectively), while the first external validation presented an even distribution of elevated, flat, and depressed lesion morphologies. Meanwhile, 63 (22.9%) and 16 (12.6%) cases had ulcers in the first and second external validation data sets, respectively. The overall rate of curative resection ranged from 36.4% (100/275) to 74.4% (2010/2703).

**Table 1:** Baseline characteristics of the included data sets.

Characteristics		Training and internal validation set (n=2703)	First external validation set (n=275)	Second external validation set (n=127)
**Sex, n (%)**				
	Male	1427 (52.8)	165 (60.0)	80 (62.9)
	Female	1276 (47.2)	110 (40.0)	47 (37.0)
Age (years), mean (SD)		65.9 (12.4)	67.8 (12.0)	64.1 (13.0)
**Longitudinal location, n (%)**				
	Lower-third	2069 (76.5)	214 (77.8)	53 (41.7)
	Mid-third	336 (12.4)	28 (10.2)	61 (48.1)
	Upper-third	298 (11.0)	33 (12.0)	13 (10.2)
**Circular location, n (%)**				
	Lesser curvature	945 (34.9)	95 (34.5)	49 (38.6)
	Greater curvature	557 (20.6)	58 (21.1)	27 (21.3)
	Posterior wall	585 (21.6)	68 (24.7)	22 (17.3)
	Anterior wall	607 (22.5)	54 (19.6)	29 (22.8)
	More than 2 areas involved	9 (0.3)	0 (0)	0 (0)
Endoscopic size of the lesion (mm), mean (SD)		21.7 (12.5)	27.9 (16.2)	21.7 (12.6)
**Morphology, n (%)**				
	Elevated	375 (13.9)	101 (36.7)	28 (22.1)
	Flat	566 (20.9)	98 (35.6)	37 (29.1)
	Depressed	1762 (65.2)	76 (27.6)	62 (48.8)
**Ulcer, n (%)**				
	Present	504 (18.6)	63 (22.9)	16 (12.6)
	None	2199 (81.4)	212 (77.1)	111 (87.4)
**Curative resection, n (%)**				
	Yes	2010 (74.4)	100 (36.4)	87 (68.5)
	No	693 (25.6)	175 (63.6)	40 (31.5)

### Internal Validation Performance

**Figure 2:**
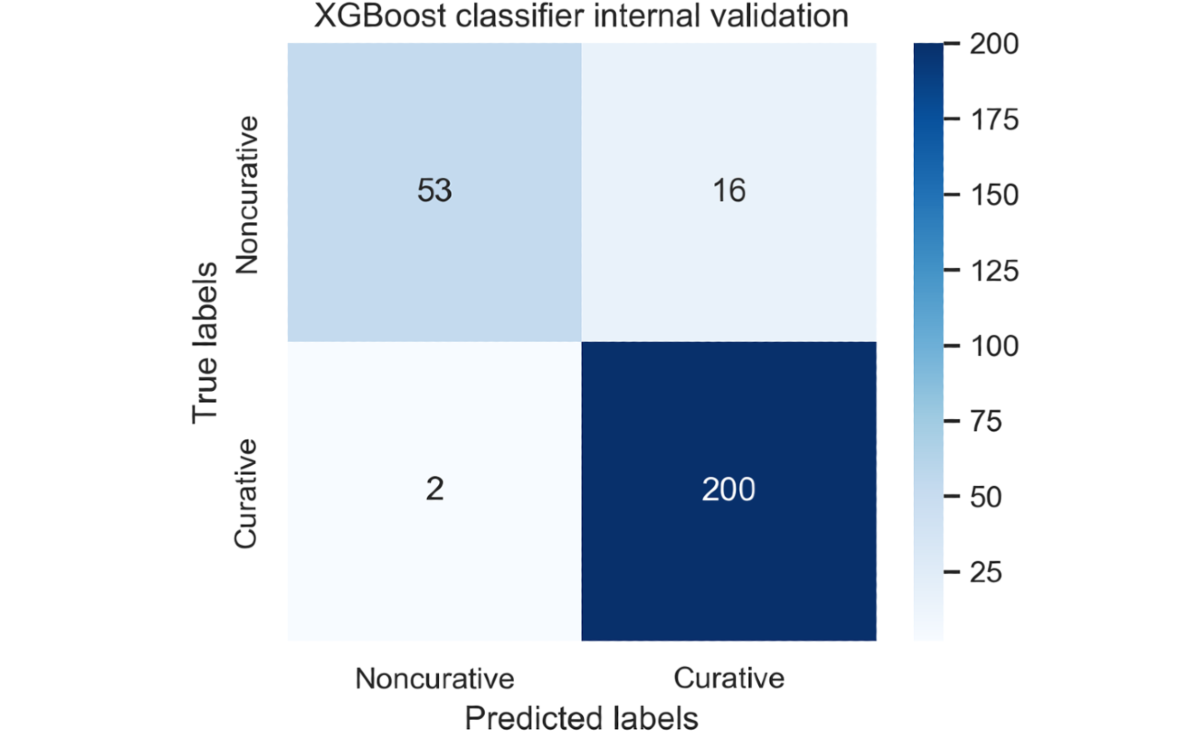
Confusion matrix for the extreme gradient boosting classifier in the internal validation cohort. XGBoost: extreme gradient boosting.

Table 2 shows the prediction performance of 18 ML classifiers for internal validation. The XGBoost classifier demonstrated the best performance as follows: internal validation accuracy 93.4%, 95% CI 90.4%-96.4%; precision 92.6%, 95% CI 89.5%–95.7%; recall 99.0%, 95% CI 97.8%-99.9%; and F1 score 95.7%, 95% CI 93.3%-98.1%. In detail, the XGBoost classifier required several parameter settings for the establishment of the ML model. The initial classifying performance of the XGBoost classifier established by us was as follows: internal validation accuracy 79.0%, 95% CI 74.1%-83.9%; precision 80.9%, 95% CI 76.2%-85.6%; recall 94.1%, 95% CI 91.3%-96.9%; and F1 score 87.0%, 95% CI 83.0%-91.0%. To discern the optimal hyperparameter setting for the establishment of the ML model, we relied on the GridSearchCV library (version 0.22) [15] to automatically search among multiple optimal parameter values to fit estimators of an ML model. By using the GridSearchCV analysis, we found the optimal hyperparameters for the best performance as follows: learning rate 0.4, maximum depth 6, and number of estimators 100. Figure 2 shows the confusion matrix for the XGBoost classifier in the internal validation data set.

**Table 2:** Internal validation performance for the prediction of curative resection of undifferentiated type of early gastric cancer by using 18 machine learning classifiers.

Machine learning classifier		Accuracy (%) (95% CI)	Precision (%) (95% CI)	Recall (%) (95% CI)	F1 score (%) (95% CI)
Gaussian Naïve Bayes		73.8 (68.6-79.0)	86.2 (82.1-90.3)	77.2 (72.2-82.2)	81.5 (76.9-86.1)
Linear discriminant analysis classifier		76.4 (71.3-81.5)	77.4 (72.4-82.4)	96.5 (94.3-98.7)	85.9 (81.8-90.0)
Logistic regression classifier		77.5 (72.5-82.5)	80.5 (75.8-85.2)	92.1 (88.9-95.3)	85.9 (81.8-90.0)
Linear support vector machine classifier		74.5 (69.3-79.7)	74.5 (69.3-79.7)	99.9 (98.8-99.9)	85.4 (81.2-89.6)
Stochastic gradient descent classifier		74.5 (69.3-79.7)	77.6 (72.6-82.6)	92.6 (89.5-95.7)	84.4 (80.1-88.7)
Decision tree classifier		74.5 (69.3-79.7)	74.5 (69.3-79.7)	99.9 (98.8-99.9)	85.4 (81.2-89.6)
k-nearest neighbors classifier		72.0 (66.7-77.3)	78.1 (73.2-83.0)	86.6 (82.5-90.7)	82.2 (77.6-86.8)
Deep neural network		77.9 (73.0-82.8)	80.6 (75.9-85.3)	92.6 (89.5-95.7)	86.2 (82.1-90.3)
**Ensemble (bagging)**					
	Bagging classifier	72.0 (66.7-77.3)	81.2 (76.5-85.9)	81.2 (76.5-85.9)	81.2 (76.5-85.9)
	Random forest classifier	72.7 (67.4-78.0)	80.2 (75.5-84.9)	84.2 (79.9-88.5)	82.1 (77.5-86.7)
	Voting classifier	84.5 (80.2-88.8)	88.1 (84.2-92.0)	91.6 (88.3-94.9)	89.8 (86.2-93.4)
**Ensemble (boosting)**					
	Gradient boosting classifier	77.5 (72.5-82.5)	80.5 (75.8-85.2)	92.1 (88.9-95.3)	85.9 (81.8-90.0)
	Adaptive boosting classifier	77.9 (73.0-82.8)	81.1 (76.4-85.8)	91.6 (88.3-94.9)	86.0 (81.9-90.1)
	Categorical boosting classifier	84.1 (79.7-88.5)	83.8 (79.4-88.2)	97.5 (95.6-99.4)	90.2 (86.7-93.7)
	Extreme gradient boosting classifier	93.4 (90.4-96.4)	92.6 (89.5-95.7)	99.0 (97.8-99.9)	95.7 (93.3-98.1)
	Light gradient boosting machine classifier	75.6 (70.6-80.8)	80.9 (76.2-85.6)	88.1 (84.2-92.0)	84.4 (80.1-88.7)
	Histogram-based gradient boosting classifier	85.2 (81.0-89.4)	84.9 (80.689.2)	97.5 (95.6-99.4)	90.8 (87.4-94.2)
Ensemble (stacking)		75.6 (70.5-80.7)	78.6 (73.7-83.5)	92.6 (89.5-95.7)	85.0 (80.7-89.3)

### External Validation Performance in the XGBoost Classifier

**Figure 3:**
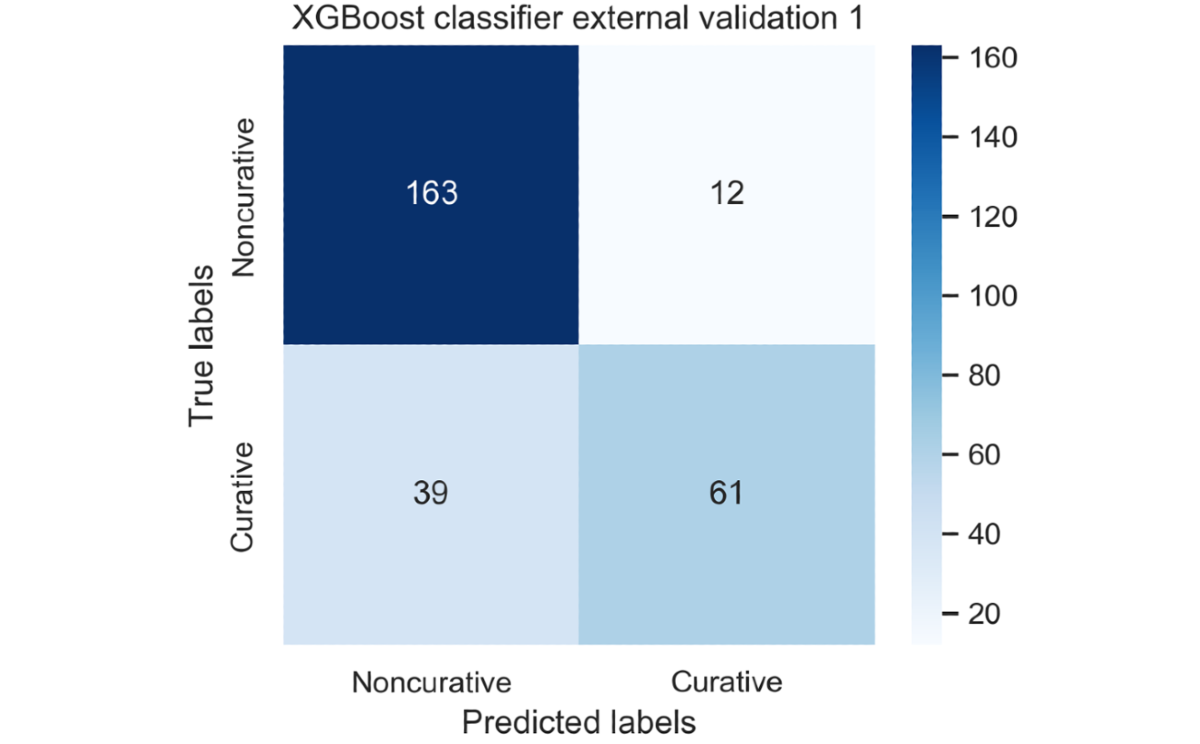
Confusion matrix for the extreme gradient boosting classifier in the first external validation cohort. XGBoost: extreme gradient boosting.

**Figure 4:**
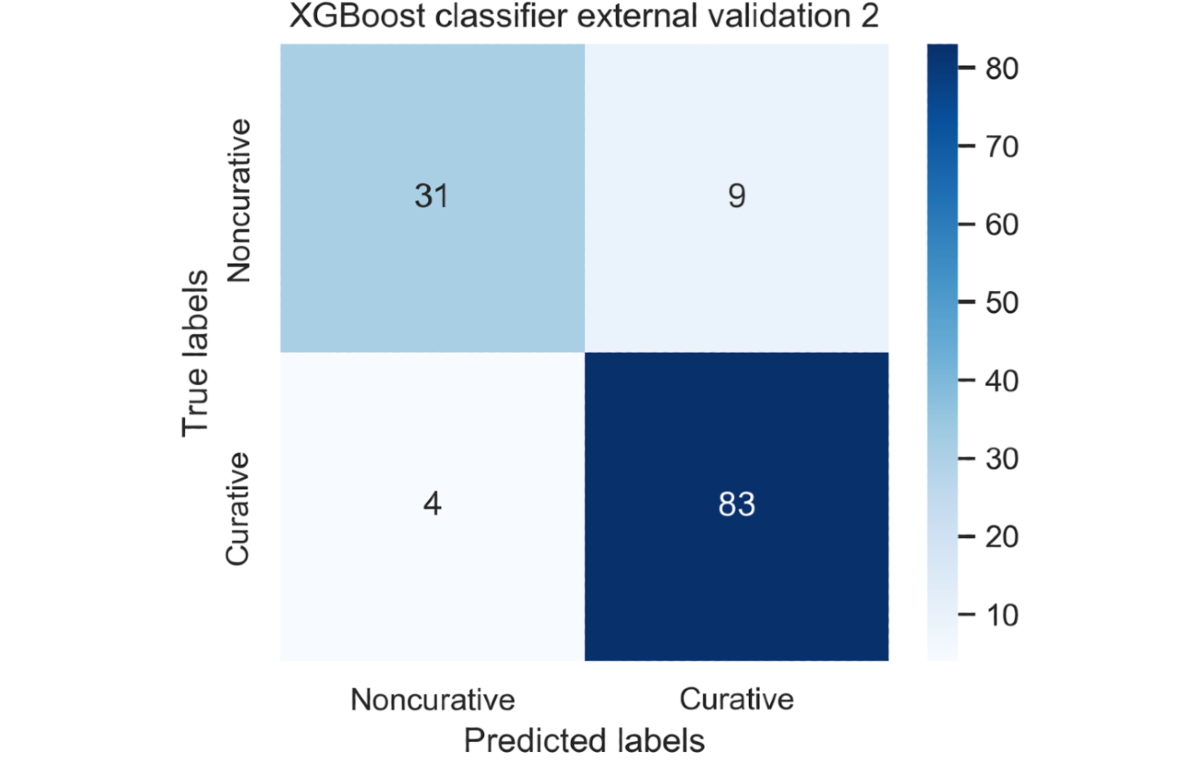
Confusion matrix for the extreme gradient boosting in the second external validation cohort. XGBoost: extreme gradient boosting.

For the first external validation data set, the XGBoost classifier demonstrated its performance as follows: external validation accuracy 81.5%, 95% CI 76.9%-86.1%; precision 83.6%, 95% CI 79.2%-88.0%; recall 61.0%, 95% CI 55.2%-66.8%; and F1 score 70.5%, 95% CI 65.1%-75.9%. Then, for the second external validation data set, the XGBoost classifier demonstrated its performance as follows: external validation accuracy 89.8%, 95% CI 84.5%-95.1%; precision 90.2%, 95% CI 85.0%-95.4%; recall 95.4%, 95% CI 91.8%-99.0%; and F1 score 92.7%, 95% CI 88.2%-97.2%. Figure 3 and Figure 4 show the confusion matrices for the XGBoost classifier in the first and second external validation data sets, respectively.

### Explainable Artificial Intelligence

Table 3 shows the univariable analysis for the associated factors of lesions determined accurately or inaccurately in the curative resection of U-EGC by the XGBoost classifier. Notably, there was no single significant factor associated with lesions determined either accurately or inaccurately by the XGBoost classifier.

**Table 3:** Univariable analysis of the associated factors of lesions determined accurately or inaccurately in the curative resection of undifferentiated type of early gastric cancer by the extreme gradient boosting classifier.

Characteristics		First external validation set			Second external validation set		
		Accurately determined by XGBoost^a^ classifier (n=224)	Inaccurately determined by XGBoost classifier (n=51)	*P* value	Accurately determined by XGBoost classifier (n=114)	Inaccurately determined by XGBoost classifier (n=13)	*P* value
**Sex, n (%)**				.06			.37
	Male	128 (57.1)	37 (73)		70 (61.4)	10 (77)	
	Female	96 (42.9)	14 (28)		44 (38.6)	3 (23)	
Age (years), mean (SD)		67.3 (12.6)	70.0 (9.2)	.09	63.9 (13.3)	65.5 (10.4)	.66
**Longitudinal location, n (%)**				.22			.33
	Lower-third	173 (77.2)	41 (80)		50 (43.9)	3 (23)	
	Mid-third	21 (9.4)	7 (14)		53 (46.5)	8 (62)	
	Upper-third	30 (13.4)	3 (6)		11 (9.6)	2 (15)	
**Circular location, n (%)**				.38			.29
	Lesser curvature	74 (33.0)	21 (41)		46 (40.4)	3 (23)	
	Greater curvature	45 (20.1)	13 (26)		23 (20.2)	4 (31)	
	Posterior wall	58 (25.9)	10 (20)		21 (18.4)	1 (8)	
	Anterior wall	47 (20.9)	7 (14)		24 (21.1)	5 (39)	
Endoscopic size of the lesion (cm), mean (SD)		28.4 (16.4)	25.5 (14.7)	.25	22.2 (12.7)	17.1 (11.4)	.16
**Morphology, n (%)**				.36			.93
	Elevated (I, IIa, and IIa+IIc)	78 (34.8)	23 (45)		25 (21.9)	3 (23)	
	Flat (IIb)	81 (36.2)	17 (33)		34 (29.8)	3 (23)	
	Depressed (IIc)	65 (29)	11 (22)		55 (48.2)	7 (54)	
**Ulcer, n (%)**				.86			.21
	Present	52 (23.2)	11 (22)		13 (11.4)	3 (23)	
	None	172 (76.8)	40 (78)		101 (88.6)	10 (77)	

^a^XGBoost: extreme gradient boosting.

**Figure 5:**
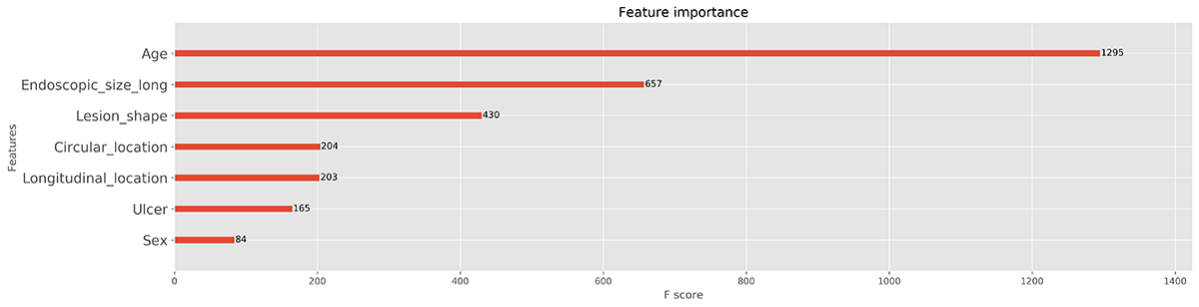
Feature importance plot for the extreme gradient boosting classifier in the internal validation cohort. The average F-score was calculated through 50 repetitions of five-fold cross-validation in the training data set.

Figure 5 shows the feature importance plot for the XGBoost classifier. Age, endoscopic size, and morphology of the lesions were the three most significant factors for the establishment of the ML model, in sequence. Multimedia Appendix 1 illustrates the decision process tree for the XGBoost classifier prior to adopting the GridSearchCV library. This simplified tree shows the step-by-step determination process of the ML model. The final leaf score is inserted in the following equation: *p(x)* = 1 / 1 + *e^–leaf score^*. Any value over 0.5 (50%) indicates curative resection and any value less than 0.5 indicates noncurative resection, as predicted by the XGBoost classifier [21]. Multimedia Appendix 2 shows the final decision process tree for the XGBoost classifier after adopting the GridSearchCV library, which presented the best performance in the internal validation. Endoscopic size of the lesion, patient age, and longitudinal location of the lesion were the important factors, in sequence. Multimedia Appendix 3 shows the partial-dependence target plot for the feature of endoscopic size of the lesion in the first external validation assessment. The probability of curative resection for the lesions with sizes ranging from 4 mm to 10 mm reached 80%. Meanwhile, U-EGC lesions with sizes ranging from 20.78 mm to 26.22 mm showed the lowest probability of curative resection at 16.1%. Multimedia Appendix 4 presents the two-way partial-dependence target plot for the features of endoscopic size of the lesion and patient age in the first external validation cohort. Given that the color of the circle above the imaginary line of Y=X is darker than that below the line, the endoscopic size and age are suggested to be correlated with curative resection of U-EGC. Multimedia Appendix 5 shows the partial-dependence interaction plot for the features of endoscopic size of the lesion and age in the first external validation group. Given that the contour lines are generally parallel to the Y-axis, the probability of curative resection is more dependent on the endoscopic size of the lesion. Since the feature importance analysis measures the prediction error after permutating the features’ values, the results can be skewed when the said features exhibit dependency. However, the Shapley value considers the influence of the features on each other. Multimedia Appendix 6 and Multimedia Appendix 7 demonstrate the summary plot and bar plot of the Shapley additive explanations analysis, respectively, where endoscopic size of the lesion and age are the important features for the model output.

## Discussion

This study introduces the good performance of an ML model applied to the prediction of curative resection of U-EGC prior to ESD, suggesting the possibility of a beneficial effect of ML modeling for decision making in this part of clinical practice [22]. Moreover, thorough external validations confirmed the higher rate of curative resection predicted by ML modeling as compared with curative resection rates reported by clinicians. To our knowledge, this is the first study to establish and confirm the predictive performance of an artificial intelligence model for the therapeutic outcomes of ESD for U-EGC. Indeed, ML is characterized as a computer-aided prediction method and its most important benefit in this context consists of the improvement in predictive accuracy for curative resection prior to ESD. The proper selection of candidates for ESD is essential before beginning ESD. The most fundamental hypothesis is that endoscopic resection can be performed with curative intent in cases of EGC without lymph node metastasis. Therefore, indications of ESD were established using a combination of factors associated with a negligible lymph-node metastasis rate from the retrospective analysis of surgically resected specimens [3]. These indications are categorized by differentiated-type EGC and U-EGC according to the differentiation, specific size, and morphological and histological conditions of the involved lesion. However, optical endoscopic determination of the factors stated above involves operator-dependent characteristics. In the study of a Korean multicenter registry of ESD for U-EGC, there was a discrepancy between pre-ESD indications and post-ESD criteria in 36.7% of all the lesions [6]. Underestimation of the size was the most common reason for noncurative resection (71.4%), followed by underestimation of the depth of invasion (32%) and unpredictability of lymphovascular invasion (14.9%) [6]. Although adopting a precise indication is important, U-EGC itself is a risk factor for an enhanced out-of-indication rate, leading to noncurative resection; therefore, more strict indications might be necessary for pursuing the ESD of U-EGC [11,12].

Another important finding of this study is the presentation of the determination reason or process of the ML model through the explainable artificial intelligence analysis. Notably, there is a tradeoff between accuracy and interpretability in the classification model of ML [14]. Although the ML approach exhibited high degrees of accuracy based on complex calculations, it is characterized by low interpretability (artificial intelligence is more generally characterized as being of a “black-box nature”) [14]. Conventional statistical analyses such as univariate or multivariate logistic regression analyses in previous studies have shown the reasons underlying the lower curative resection rate of ESD for U-EGC [5,6]. However, there is a limitation in the explanatory power of the overall model (low accuracy) in these studies. The XGBoost classifier used parallel-tree boosting analysis to provide highly efficient and accurate predictions. Through the ensemble model and extensive explainable artificial intelligence analysis, we identified the size of the lesion as being the most important feature for the successful prediction of curative resection in the ESD of U-EGC. Although a prospective trial of ESD for U-EGC that satisfied the expanded indication reported an excellent long-term survival rate [6,23,24], more cautious application or restriction of ESD indications has been recommended, especially regarding the size categorization [3,25]. Most recently published studies have also indicated that small intramucosal U-EGC lesions measuring less than 1.0 cm or 1.5 cm without lymphovascular invasion should be considered as the ESD candidate [26,27]. The explainable artificial intelligence analysis in our study also revealed that U-EGC lesions of less than 1 cm have the greatest probability of curative resection (Multimedia Appendix 3). Considering that the aim of this study was not the validation of current ESD criteria, further studies with robust analysis would elucidate the value of these findings.

In the context of ecological factors, age and gender have been tested with the endoscopic factors for the potential variable for the curative resection rate prediction. However, these variables were not consistently identified as important indicators for predicting curative resection [28-30]. Although feature importance analysis (Figure 5) or Shapley additive explanations analysis (Multimedia Appendix 6) in our study revealed that age is an important variable for the ML determination process, explainable artificial intelligence analysis is currently an experimental method to understand how ML judges. It is presumed that the reason ML shows higher accuracy than traditional statistics is that it performs a complex operation that considers all variables. It is true that age is an important factor influencing ML judgment, but further explainable artificial intelligence statistics can explain how much it affects the actual curative resection.

Although this study established and rigorously validated the predictive performance of the designed ML model, several inevitable limitations became apparent. First, there was some discrepancy in the validation performance between the first and second external data sets. The indications of ESD for U-EGC have not been approved by all endoscopists. Therefore, practice patterns adopting ESD indications for U-EGC have been heterogenous depending on the institution. The first external validation data set was more heterogenous with respect to the baseline characteristics and therapeutic outcomes. However, the second data set was collected from a single institution, thus providing a more discrete application pattern of the ESD indication for U-EGC. Second, patient age was an important feature in the explainable artificial intelligence analysis; however, this feature does not perfectly reflect the general condition of the patient. Further, there is no age factor for ESD indications. However, the general condition of the patients is frequently considered in the determination of whether to pursue ESD. Therefore, clinical factors that reliably reflect patients’ health status other than age should be developed and considered so as to attain the most favorable therapeutic outcomes of ESD. Third, the training and internal validation data sets included cases that were surgically resected as well as endoscopically resected cases. Endoscopists decide whether to perform ESD or surgery when they detect U-EGC. In other words, it has not been determined which U-EGC is a candidate for ESD or surgery. All the U-EGCs resected with surgery or ESD were included as it was not always accurate and appropriate for the endoscopists to differentiate between ESD or surgery. If only U-EGCs that were resected by ESD were collected, a clear ESD candidate would have been collected, which in itself may be a selection bias. In conclusion, we established an ML model capable of accurately predicting the curative resection of U-EGC prior to ESD by considering the morphological and ecological characteristics of the lesions. A clinical application study in a randomized controlled manner would elucidate the real value of this ML model.

## Appendix

Multimedia Appendix 1 Decision process tree for the extreme gradient boosting classifier prior to adopting the GridSearchCV library in the internal validation assessment.

Multimedia Appendix 2 Decision process tree for the extreme gradient boosting classifier after adopting GridSearchCV library in the internal validation assessment.

Multimedia Appendix 3 Partial-dependence target plot for the feature of endoscopic size of the lesion in the first external validation cohort.

Multimedia Appendix 4 Two-way partial-dependence target plot for the features of endoscopic size of the lesion and patient age in the first external validation cohort.

Multimedia Appendix 5 Partial-dependence interaction plot for the features of endoscopic size of the lesion and patient age in the first external validation cohort.

Multimedia Appendix 6 Summary plot of the Shapley additive explanations analysis. Endoscopic size of the lesion and patient age are the important features for the model output.

Multimedia Appendix 7 Bar plot of the Shapley additive explanations analysis. Endoscopic size of the lesion and patient age are the important features for the model output.

## Abbreviations

EGC: early gastric cancer
ESD: endoscopic submucosal dissection
ML: machine learning
U-EGC: undifferentiated type of early gastric cancer
XGBoost: extreme gradient boosting
