# Supplementary material for: Establishing Machine Learning Models to Predict Curative Resection in Early Gastric Cancer with Undifferentiated Histology: Development and Usability Study
Source: J Med Internet Res. 2021 Apr 15;23(4):e25053. doi: 10.2196/25053 (PMC8085749; doi:10.2196/25053)
Supplement: Multimedia Appendix 2 [file jmir_v23i4e25053_app2.docx]

**Multimedia Appendix 2**

Decision process tree for the XGBoost classifier after adopting GridSearchCV library in the internal-validation assessment.


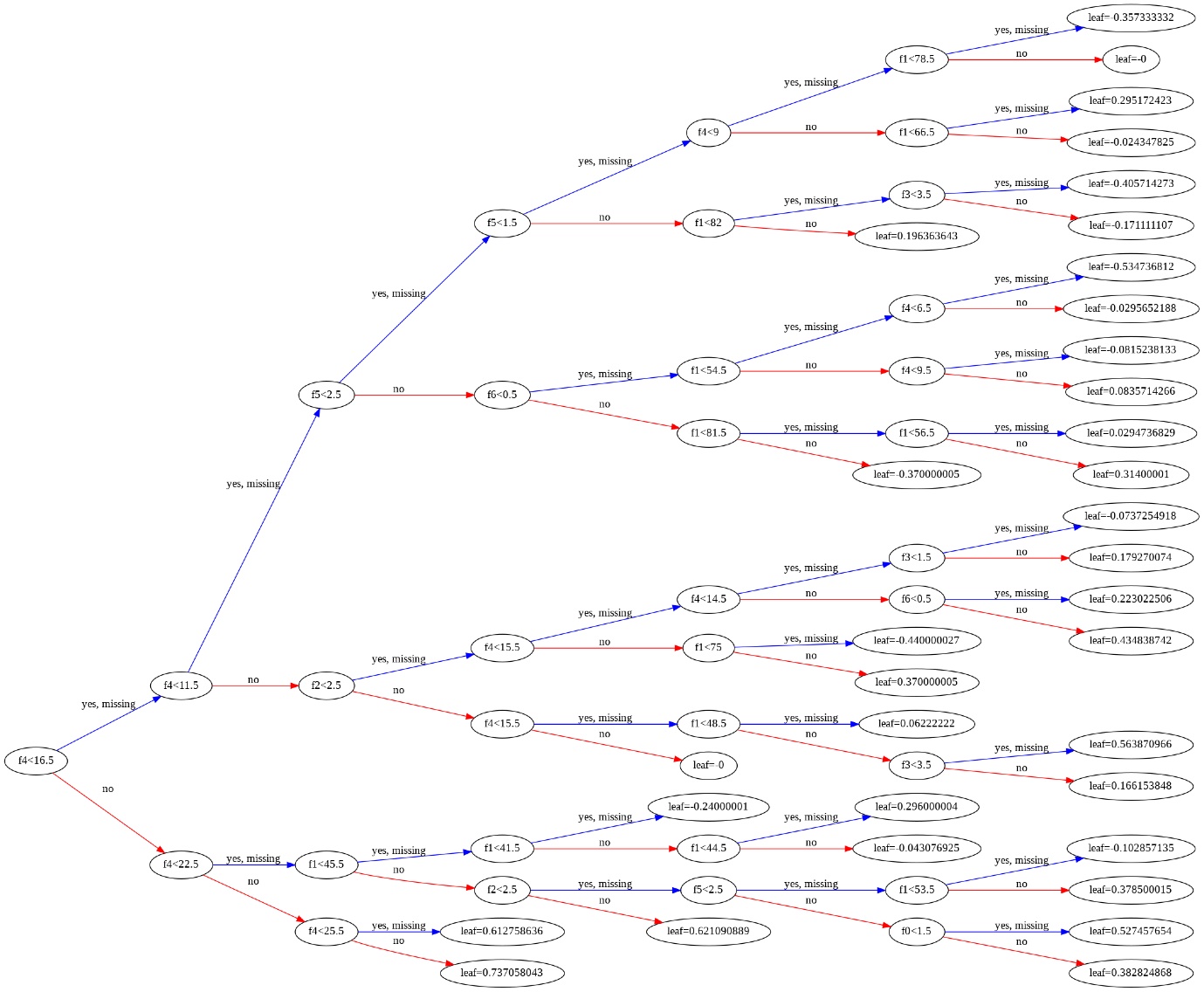


f4: endoscopic size of the lesion, f1: age, f2: longitudinal location of the lesion, f5: morphology of the lesion, f6: ulcer, f3: circular location of the lesion, f0: sex, XGBoost, extreme gradient boosting.
