# Supplementary material for: Establishing Machine Learning Models to Predict Curative Resection in Early Gastric Cancer with Undifferentiated Histology: Development and Usability Study
Source: J Med Internet Res. 2021 Apr 15;23(4):e25053. doi: 10.2196/25053 (PMC8085749; doi:10.2196/25053)
Supplement: Multimedia Appendix 3 [file jmir_v23i4e25053_app3.docx]

**Multimedia Appendix 3**

Partial-dependence target plot for the feature of endoscopic size of the lesion in the first external-validation cohort.


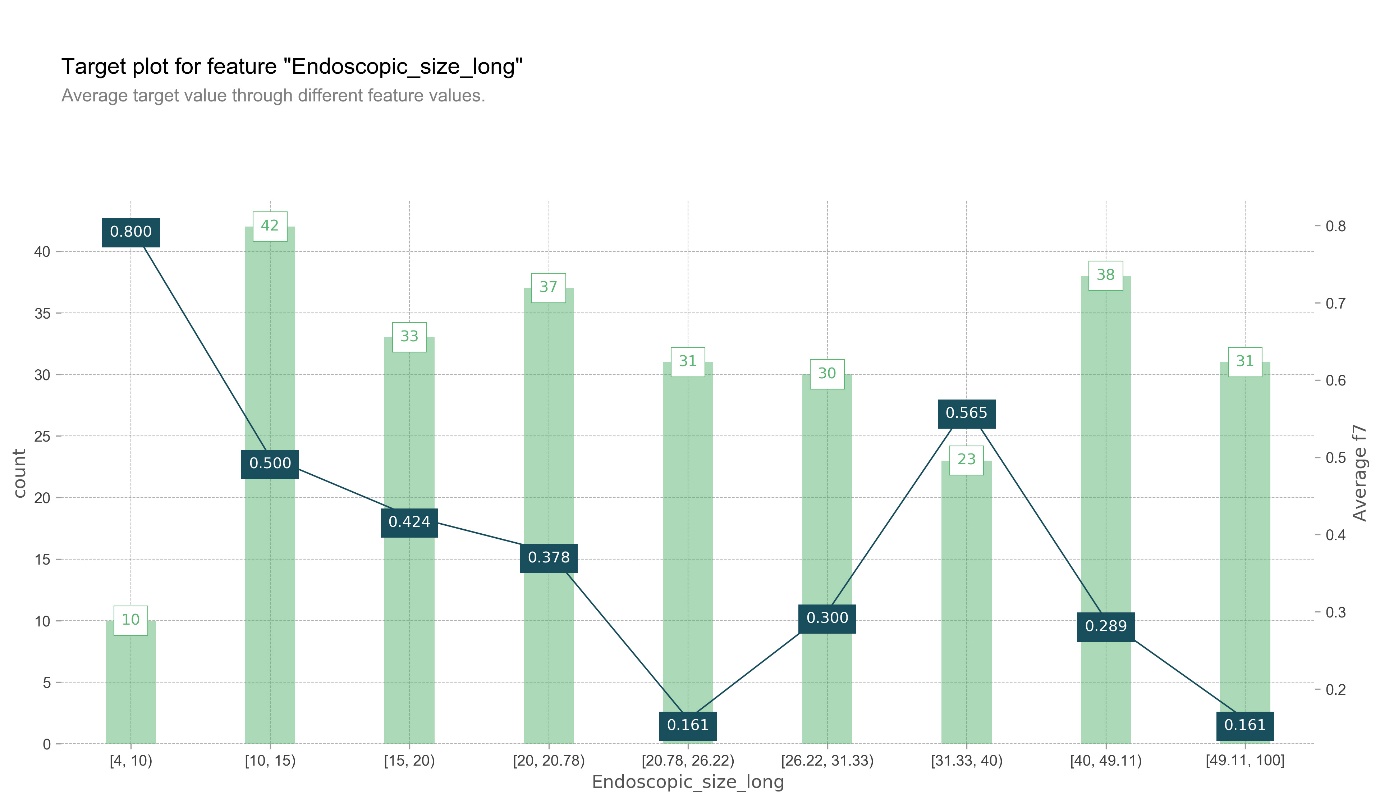


The value in the bar graph indicates the crude number of lesions in each endoscopic size category. The value in the line graph indicates the probability of curative resection for the lesions in each endoscopic size category.
