# Supplementary material for: Establishing Machine Learning Models to Predict Curative Resection in Early Gastric Cancer with Undifferentiated Histology: Development and Usability Study
Source: J Med Internet Res. 2021 Apr 15;23(4):e25053. doi: 10.2196/25053 (PMC8085749; doi:10.2196/25053)
Supplement: Multimedia Appendix 4 [file jmir_v23i4e25053_app4.docx]

**Multimedia Appendix 4**

Two-way partial-dependence target plot for the features of endoscopic size of the lesion and patient age in the first external-validation cohort.


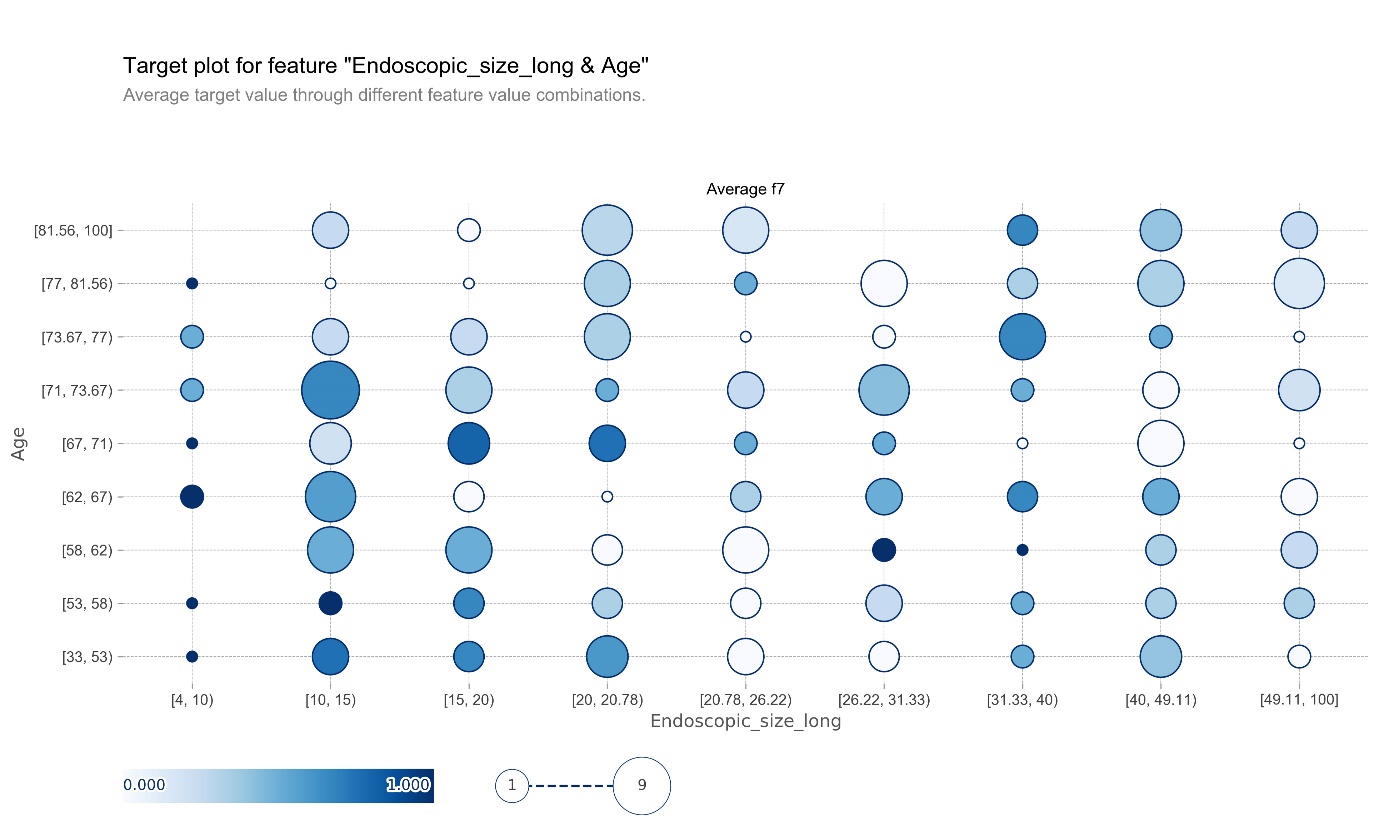


The size of the circle indicates the crude number of lesions in each feature-specific category. The darker blue color suggests a higher probability of curative resection as compared with the lighter blue color for the lesions in each feature-specific category.
