# Supplementary material for: Establishing Machine Learning Models to Predict Curative Resection in Early Gastric Cancer with Undifferentiated Histology: Development and Usability Study
Source: J Med Internet Res. 2021 Apr 15;23(4):e25053. doi: 10.2196/25053 (PMC8085749; doi:10.2196/25053)
Supplement: Multimedia Appendix 5 [file jmir_v23i4e25053_app5.docx]

**Multimedia Appendix 5**

Partial-dependence interaction plot for the features of endoscopic size of the lesion and patient age in the first external-validation cohort.


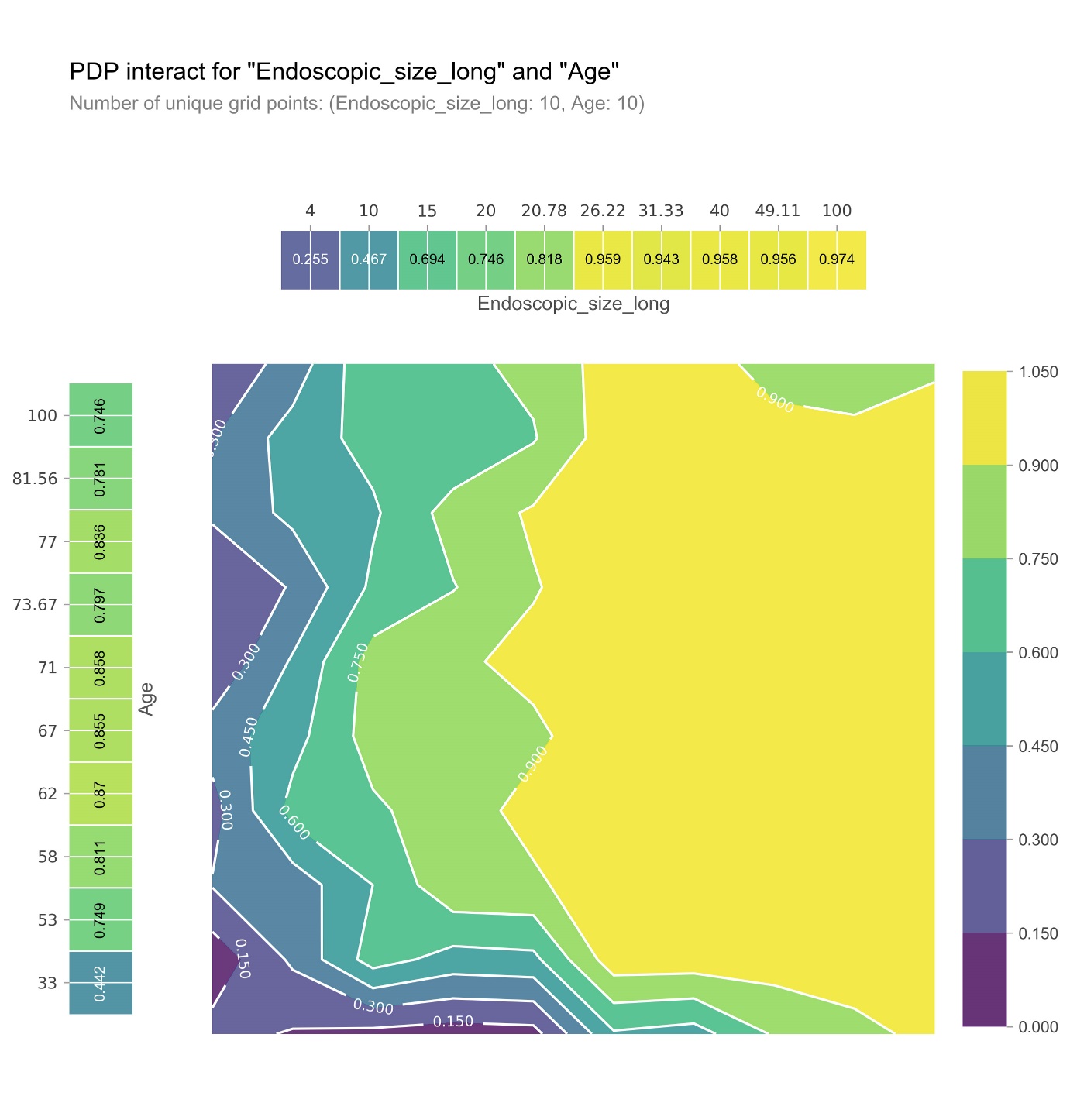


The contour lines are parallel to the Y-axis; the probability of curative resection is more dependent on the endoscopic size of the lesion.
